# C-reactive protein does not opsonize early apoptotic human neutrophils, but binds only membrane-permeable late apoptotic cells and has no effect on their phagocytosis by macrophages

**DOI:** 10.1186/1476-9255-2-5

**Published:** 2005-05-31

**Authors:** Simon P Hart, Karen M Alexander, Shonna M MacCall, Ian Dransfield

**Affiliations:** 1 MRC Centre for Inflammation Research, University of Edinburgh Medical School, Teviot Place, Edinburgh EH8 9AG, UK

## Abstract

**Background:**

It has been reported that C-reactive protein (CRP) binds both leukocyte FcγRIIA (CD32) and the plasma membrane of apoptotic cells. Since FcγRIIA becomes functionally enabled during neutrophil apoptosis, we sought to determine whether CRP bound to apoptotic neutrophils via FcγRIIA.

**Methods:**

We prepared directly labelled CRP and demonstrated that it was essentially free of IgG. We looked for evidence of CRP binding to intact, membrane impermeable apoptotic human neutrophils and to FcγRIIA-transfected Jurkat cells. We examined the functional consequences of incubation with CRP upon phagocytosis of apoptotic cells by human monocyte-derived macrophages.

**Results:**

We could not detect binding of purified soluble CRP to classical early apoptotic human neutrophils or to FcγRIIA-transfected Jurkat cells. In contrast, membrane-permeable late apoptotic neutrophils exhibited strong CRP binding, which comprised both Ca^2+^-dependent and heparin-inhibitable Ca^2+^-independent components. However, there was no effect of CRP binding upon phagocytosis of late apoptotic neutrophils by macrophages.

**Conclusion:**

Potential apoptotic cell opsonins such as CRP may bind only to intracellular structures in cells with leaky membranes that have progressed to a late stage of apoptosis.

## Background

In acute inflammation huge numbers of neutrophils are recruited to sites of tissue injury where they die by undergoing apoptosis [1]. Macrophage clearance of apoptotic neutrophils has been studied extensively under serum-free conditions in vitro, but the presence of opsonins in the inflammatory milieu means it is unlikely that "naked" apoptotic cells would be encountered by macrophages *in vivo *[2]. We have recently reported that IgG-containing immune complexes bound preferentially to functionally enabled FcγRIIA (CD32) on apoptotic neutrophils [3,4]. It has been proposed that FcγRIIA is also a receptor for the pentraxin C-reactive protein (CRP) [5,6], serum concentrations of which may increase more than 1000-fold during acute inflammation [7,8]. Independently, it was reported that soluble CRP opsonised apoptotic Jurkat cells *in vitro *[9-11]. In the present study we sought to determine whether binding of CRP to apoptotic neutrophils was mediated by FcγRIIA. To circumvent the inherent difficulties in interpreting the results of antibody binding to Fc receptor-bearing cells, we used directly fluorescein-conjugated purified CRP that was essentially free of contaminating IgG.

## Methods

### Conjugation of CRP with FITC

1 mg of CRP purified from human plasma (Sigma; Poole, UK) was dissolved in 1 ml deionised water and dialysed against 100 mM sodium bicarbonate pH 8.25. Fluorescein isothiocyanate (FITC; Sigma) was dissolved at 1.5 mg/ml in DMSO and added dropwise to a total volume of 45 μl per ml of protein solution. The mixture was incubated for 2 hours at room temperature in the dark. Unconjugated FITC was removed by exhaustive dialysis.

### Assessment of CRP purity

Native and FITC-conjugated CRP were examined under denaturing conditions on a 9% acrylamide gel stained with Coomassie blue. Contamination with human IgG was assessed by comparing Western blots of CRP with known amounts of human IgG (Sigma). Blots were probed with rabbit F(ab')_2 _anti-human IgG followed by peroxidase-conjugated goat anti-rabbit IgG (DakoCytomation; Ely, UK) and developed by enhanced chemiluminescence (Amersham).

### CRP phospholipid binding assay

One micrometer diameter polystyrene microspheres (Polysciences; Warrington, PA) were coated with 1 mg/ml phosphorylcholine-conjugated BSA (Biosearch Technologies; Novato, CA) or BSA (Sigma) in PBS for 1 h at room temperature. Beads were washed and incubated with FITC-conjugated CRP in the presence of 2 mM Ca^2+ ^or 5 mM EDTA. FITC-CRP binding was measured by gating on single beads and analysing fluorescence in the FL1 channel (530 nm) following excitation with an argon laser at 488 nm in a BD FACSCalibur flow cytometer (BD Biosciences, Cowley, Oxford, UK).

### CRP binding assay

Human neutrophils were isolated from peripheral blood of healthy volunteers by dextran sedimentation and discontinuous Percoll gradient centrifugation [12]. Genomic DNA extraction and determination of the polymorphism at position 519 in exon 4 of the FcγRIIA gene was performed as previously described [3]. G or A at position 519 leads to either an arginine (R) or histidine (H) amino acid at position 131 in the second Ig-like domain of the FcγRIIA protein. Experiments were performed using cells from donors with each of the genotypes R/R, R/H, and H/H. Neutrophils were aged in culture for 20 h at 37°C/5% CO_2 _in Iscove's medium (Invitrogen, Paisley, UK) containing 10% autologous serum or FCS. Jurkat cells transfected with human FcγRIIA or control vector were kindly provided by Dr. Eric Brown, University of California, San Francisco, USA [13]. Surface phenotyping using indirect immunofluorescence and flow cytometry confirmed expression of FcγRIIA by the transfected cells, but no expression of FcγRI or FcγRIII was detected. Control Jurkat cells transfected with empty vector did not express any Fc receptors. Cells were washed twice in PBS prior to use. Cell binding assays were performed in 140 mM NaCl pH 7.4, 20 mM HEPES, and either 2 mM CaCl_2 _or 5 mM EDTA. FITC-CRP was incubated with cells for 30 minutes on ice, then washed twice in binding buffer and incubated with phycoerythrin-conjugated Annexin V (Caltag; Towchester, UK) or 5 μg/ml propidium iodide. In some experiments cells were incubated with 1 mg/ml unfractionated heparin (Sigma) and washed twice prior to incubation with FITC-CRP. Fluorescence was analysed on an Coulter Epics XL flow cytometer (Beckman Coulter, High Wycombe, UK) and/or a BD FACSCalibur flow cytometer.

### Immunofluorescence microscopy

Aged neutrophils were labelled with 50 μg/ml FITC-CRP, fixed in 3% paraformaldehyde, permeabilised with 0.1% Triton X-100, counterstained with TO-PRO-3 (Molecular Probes, Leiden, NL), and cytocentrifuged onto glass slides. Visualisation was performed with a Leica TCSNT confocal system (Leica Microsystems, GmBH, Mannheim, Germany).

### Flow cytometric cell sorting

Cultured human neutrophils were labelled with 25 μg/ml FITC-CRP and sorted according to FL1 signal intensity using a BD FACSVantage fluorescence activated cell sorter (FACS). Sorted cell populations were checked for purity by flow cytometry, and cell morphology was examined on May-Giemsa-stained cytocentrifuge preparations.

### Macrophage phagocytosis of late apoptotic neutrophils

Human neutrophils were labelled with CFDA (CellTracker™Green; Molecular Probes) and incubated at 37°C for 72 h in Iscove's medium containing 10% autologous serum to yield a cell population that contained >70% late apoptotic neutrophils. Aged neutrophils were washed, and incubated with 100 μg/ml CRP or Iscove's medium alone for 30 minutes. Monolayers of 5–8d old human monocyte-derived macrophages in 48 well plates were incubated with 2 × 10^6 ^aged neutrophils in Iscove's medium in the absence of serum for 60 minutes at 37°C [14]. The supernatant was aspirated and the macrophages were detached by brief incubation in 0.05% trypsin-0.02% EDTA (Invitrogen) and vigorous pipetting. The percentage of macrophages that had ingested one or more apoptotic neutrophils was determined by flow cytometric analysis as previously described [15].

### Statistical analysis

Results are presented as mean ± SEM of at least three independent experiments using cells from different donors. Results were compared using either a paired t-test or repeated measures ANOVA and Tukey-Kramer multiple comparisons test as appropriate, using GraphPad InStat version 3 (GraphPad Software, San Diego, CA).

## Results

### Purity and functional integrity of FITC-conjugated CRP

**Figure 1:**
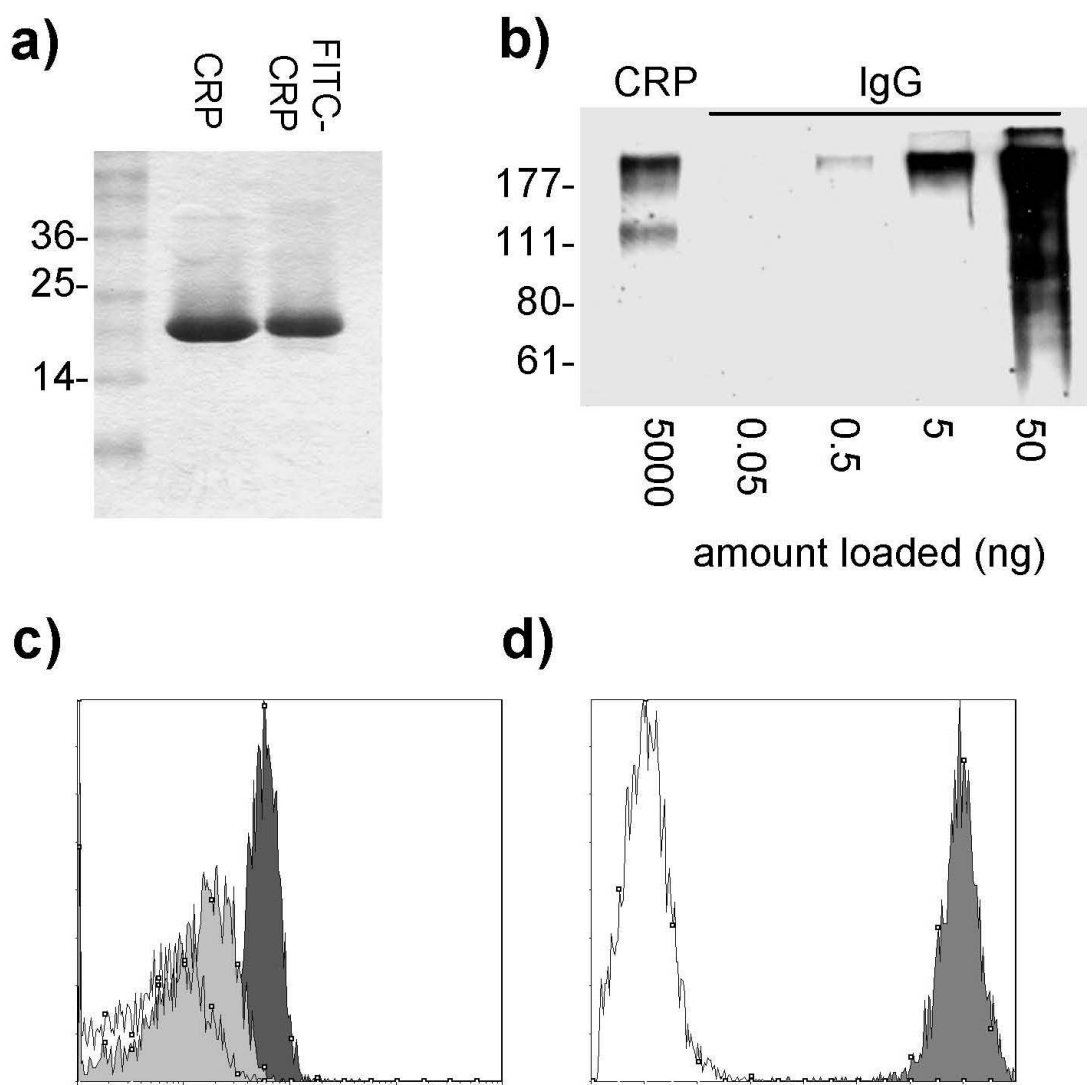
**Purity and functional integrity of FITC-CRP**. (a) Native and FITC-conjugated CRP migrated as single bands of approximately 23 kD in SDS-PAGE. (b) Western blot of CRP and known amounts of human IgG on a 9% acrylamide gel under non-reducing conditions. The blot was probed with anti-human IgG-HRP. CRP contained <0.1% IgG (w/w). MW markers in kDa are illustrated. (c) FITC-CRP bound to phosphorylcholine-coated beads in the presence of 2 mM Ca^2+ ^(dark shaded histogram). Binding in 5 mM EDTA (light shaded histogram) and BSA-coated beads (control; open histogram) is also shown. (d) FITC-CRP bound to freeze-thawed necrotic neutrophils (shaded histogram). Binding of FITC-BSA is shown as a control (open histogram).

Binding studies were performed with human plasma-derived CRP that had been directly conjugated to FITC. Native CRP and FITC-CRP migrated as single bands when examined by SDS-PAGE (Figure 1a). Western blotting demonstrated <0.1% contamination of our CRP preparation with human IgG. (Figure 1b). FITC-CRP was shown to be functionally active by demonstrating Ca^2+^-dependent binding to phosphorylcholine-coated beads (Figure 1c). Similarly, FITC-CRP bound strongly to cells that had been rendered necrotic by freeze-thawing (Figure 1d).

### CRP does not bind to early apoptotic neutrophils

**Figure 2:**
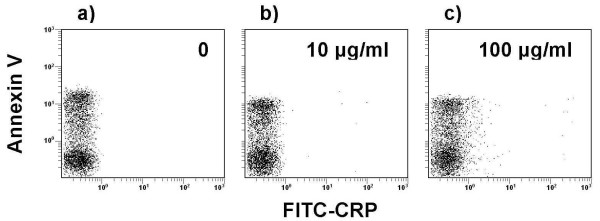
**Lack of CRP binding to early apoptotic human neutrophils**. FITC-CRP binding to aged human neutrophils was assessed by dual color flow cytometry using annexin V-PE to identify apoptotic cells. In comparison with buffer alone (a), FITC-CRP did not bind to apoptotic neutrophils at concentrations up to 100 μg/ml (b,c).

Early apoptotic neutrophils were identified within a population of cultured human neutrophils by their characteristic flow cytometric laser scatter properties, labelling with annexin V, and exclusion of propidium iodide. We found no detectable binding of CRP at concentrations up to and including 100 μg/ml, in presence or absence of 2 mM Ca^2+ ^(Figure 2). The highest concentration of CRP that we used is comparable to serum concentrations recorded during an acute inflammatory response. The lack of CRP binding was observed regardless of FcγRIIA genotype (data not shown).

### Lack of binding of CRP to FcγRIIA

**Figure 3:**
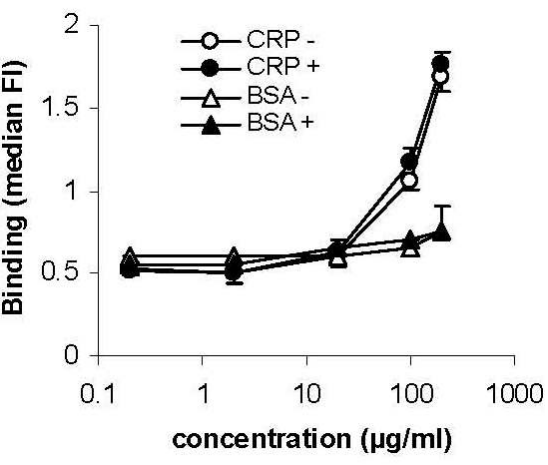
**CRP does not bind to FcγRIIA**. Jurkat cells transfected with empty vector (-; open symbols) or human FcγRIIA (+; closed symbols) were incubated with FITC-conjugated CRP (circles) or FITC-BSA control (triangles). There was no diffrence in binding between transfected and non-transfected cells.

The lack of binding of CRP to non-apoptotic or apoptotic neutrophils suggested that soluble (non-aggregated) CRP was unable to bind to FcγRIIA with significant affinity. For confirmation, Jurkat cells transfected with human FcγRIIA were incubated with FITC-conjugated CRP. There was no evidence of preferential binding of CRP to FcγRIIA-transfected cells compared with those transfected with control vector (Figure 3).

### CRP binds strongly to a subpopulation of cultured human neutrophils

**Figure 4:**
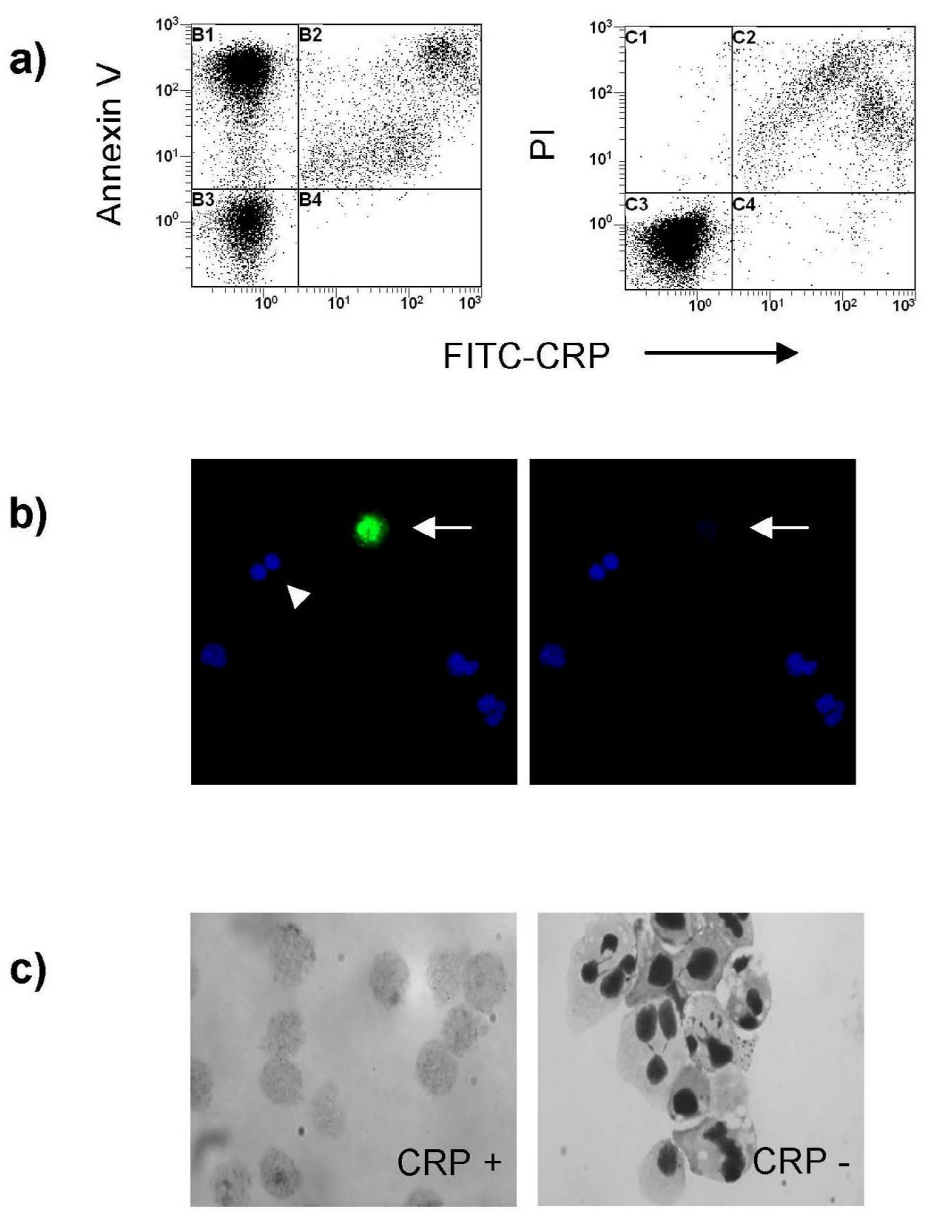
**CRP binds to a subpopulation of apoptotic neutrophils**. (a) Three color flow cytometry demonstrated that FITC-CRP (50 μg/ml) bound strongly to a subpopulation of apoptotic neutrophils that also stained with propidium iodide (PI). (b) Immunofluorescence microscopy of aged human neutrophils revealed strong CRP binding (green) to a late apoptotic neutrophil (arrow)(left panel). Nuclei have been stained with TO-PRO-3 (blue). The late apoptotic cell has almost no residual nuclear staining (right panel). There was no detectable binding to an early apoptotic neutrophil (arrowhead) or to non-apoptotic neutrophils. (c) Light microscopy of CRP^high ^(left panel) and CRP^low ^(right panel) cells sorted by FACS from a population of aged neutrophils illustrates the ghost-like morphology of CRP-binding apoptotic neutrophils. The CRP^low ^cells comprise a mixture of non-apoptotic and early apoptotic neutrophils.

A subpopulation of strongly positive cells was apparent when FITC-CRP binding to ungated cultured neutrophils was analysed. These cells were also positive for annexin V and propidium iodide staining (Figure 4a). Similar results were seen with human peripheral blood lymphocytes that had been induced to undergo apoptosis by exposure to ultraviolet radiation (data not shown). Immunofluorescence microscopy of cultured human neutrophils revealed strong CRP binding to cells that had very little or no residual nuclear staining (Figure 4b). In keeping with the flow cytometric data, there was no detectable binding to classical early apoptotic neutrophils or to non-apoptotic neutrophils. To confirm the morphology of the CRP-positive cells, cultured human neutrophils were incubated with FITC-CRP and sorted in a fluorescence-activated cell sorter (FACS). Light microscopic examination of both cell populations confirmed that the CRP^high ^cells were "ghosts" with little or no evidence of nuclear staining, whereas the CRP^low ^cells comprised a mixture of non-apoptotic and early apoptotic neutrophils (Figure 4c). The CRP^high ^neutrophils appear to have progressed to a late stage of apoptosis and undergone "nuclear evanescence" [16,17].

### Mechanism of CRP binding

**Figure 5:**
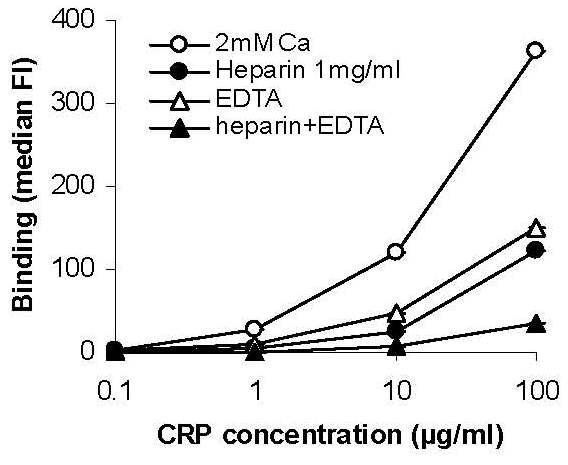
**Additive inhibition of CRP binding by EDTA and heparin**. FITC-CRP was incubated with cultured human neutrophils in the presence of 2 mM Ca^2+ ^or 5 mM EDTA, with or without pre-incubation with 1 mg/ml heparin. Binding to late apoptotic neutrophils was assessed by gating on the propidium iodide-positve cell population. The inhibitory effects of EDTA and heparin on CRP binding were additive.

CRP binding to phospholipids is dependent on the presence of calcium ions [18], whereas binding to polycationic sites is Ca^2+^-independent [19]. To determine whether Ca^2+ ^was required for CRP binding to late apoptotic neutrophils we compared FITC-CRP binding in the presence of 2 mM Ca^2+ ^and 5 mM EDTA. In the presence of EDTA, CRP binding was reduced by approximately 50% compared with total CRP binding seen in the presence of Ca^2+ ^(Figure 5). Because it has been reported that heparin binds to necrotic Jurkat cells [20], we sought to identify whether heparin and CRP bound to similar intracellullar sites. Pre-incubation with unfractionated heparin inhibited FITC-CRP binding to late apoptotic human neutrophils in the presence of cations by approximately 50%, and almost abolished residual binding when CRP was incubated in the presence of EDTA (Figure 5), suggesting that CRP bound to a combination of Ca^2+^-dependent and heparin-inhibitable Ca^2+^-independent sites.

### Macrophage phagocytosis of late apoptotic neutrophils

**Figure 6:**
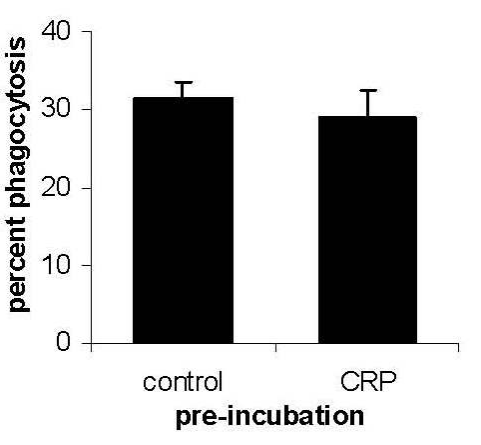
**Macrophage phagocytosis of late apoptotic neutrophils**. CRP (100 μg/ml) was allowed to bind to human neutrophils that had been aged for 72 h (>70% late apoptotic) prior to assessment of phagocytosis by human macrophages. Prior incubation with CRP had no effect on the precentage of macrophages that phagocytosed one or more late apoptotic neutrophils.

We sought to determine the effect of prior binding of CRP on phagocytosis of late apoptotic neutrophils. Our attempts to sort large numbers of cultured neutrophils by FACS into early and late apoptotic populations were unsuccessful, because during the time required for sorting many early apoptotic neutrophils progressed to late apoptosis, rendering the cells unsuitable for subsequent phagocytosis assays. Thereafter, we prepared neutrophils containing predominantly late apoptotic cells (>70%) by aging neutrophils for 72 h *in vitro*. Prior incubation of these late apoptotic neutrophils with 100 μg/ml CRP, which resulted in high levels of CRP binding, had no demonstrable effect on their phagocytosis by macrophages (Figure 6).

## Discussion

It has been reported that the pentraxins CRP and serum amyloid P are ligands for leukocyte Fcγ receptors [5]. FcγRIIA becomes functionally enabled on early apoptotic human neutrophils [3,4], but we have demonstrated that soluble CRP does not bind to classical early apoptotic human neutrophils *in vitro*, and we have been unable to demonstrate CRP binding to human FcγRIIA on transfected Jurkat cells. In the present study we have been careful to avoid pitfalls associated with indirect detection of ligand binding to neutrophils by using a preparation of directly labelled CRP that we have shown was essentially free of contaminating IgG, but which was structurally and functionally intact. The failure of CRP to bind FcγRIIA is consistent with the results of Hundt and colleagues who failed to find specific receptors for CRP on human leukocytes [6].

There have been several reports of Ca^2+^-dependent opsonisation of apoptotic cells by pentraxins [9,11,21]. The lack of CRP binding to classical early apoptotic human neutrophils, which exhibit all the biochemical and surface changes associated with apoptosis yet remain intact and membrane impermeable, raises questions about whether putative opsonins are really able to bind with high affinity to apoptotic cells. In contrast, we demonstrated very strong CRP binding to a subpopulation of aged neutrophils which displayed the characteristics of late apoptotic neutrophils previously reported by Hebert [16] and Ren [17]. It has been recognised that CRP binds to necrotic cells since Kushner and Kaplan demonstrated CRP deposition in necrotic skeletal muscle fibres following typhoid vaccination *in vivo *[22]. It is not always recognised that induction of apoptosis in many cell types *in vitro *leads to a significant proportion of membrane-permeable late apoptotic cells [23]. The presence of leaky late apoptotic cell ghosts in a cell population may be overlooked because the lack of nuclear material means that they stain very faintly with May-Giemsa, and in the past late apoptotic cells may have been gated out as "debris" when analysed by flow cytometry. Furthermore, these cells pellet poorly in cytocentrifuge preparations, and this combined with their "invisibility" with May-Giemsa stains means that their prevalence has been underestimated. This clearly has implications for studies of apoptotic cell opsonisation, and much of the published data may reflect binding to intracellular moieties. CRP is not unique in binding to the interior of leaky apoptotic cells, and we have reported a similar phenomenon with the unrelated serum protein thrombospondin [24]. Complement proteins, collectins, and heparin may also bind preferentially to late apoptotic cells [20,25,26]. The precise structures responsible for CRP binding have not been elucidated, but our data suggest that there are both Ca^2+^-dependent and Ca^2+^-independent binding sites. Binding to cell membrane phospholipids may account for the Ca^2+^-dependent component [18], whereas binding to polycations may be responsible for the heparin-inhibitable Ca^2+^-independent component [19]. The relative paucity of nuclear chromatin in the late apoptotic cells and the absence of nuclear co-localisation seen with fluorescence microscopy means that chromatin binding is unlikely to be responsible [21].

The presence of late apoptotic cells also has implications for studies of the phagocytosis of apoptotic cells, since these cells may be recognised differently from classical early apoptotic cells [17,23]. It is not known whether "opsonins" bound to intracellular components would be accessible for recognition by phagocyte receptors. In the present study we have shown that despite very strong CRP binding to late apoptotic neutrophils, there was no detectable effect on their clearance by macrophages. Ren and colleagues demonstrated that the efficiency of phagocytosis of early- and late apoptotic neutrophils was similar [17], so we think it is unlikely that an effect of CRP on uptake of late apoptotic cells has been masked by baseline uptake of early apoptotic neutrophils in the population of aged cells that we used.

## Conclusion

By using a directly labelled pure preparation of CRP we have found no evidence that CRP opsonises classical early apoptotic neutrophils *in vitro*. Like other proteins and sugars it binds intracellularly to membrane-permeable cells, but has no significant influence their subsequent phagocytosis by macrophages. A precise role for CRP remains to be elucidated.

## Abbreviations

CFDA, 5-chloromethylfluorescein diacetate; CRP, C-reactive protein; FACS, fluorescence-activated cell sorter

## Competing interests

The author(s) declare that they have no competing interests.

## Authors' contributions

SH designed the study, carried out the binding and phagocytosis experiments, analysed the data, and drafted the manuscript. KA carried out the binding and phagocytosis experiments. SM performed the cell sorting. ID participated in the design and execution of the study and drafted the manuscript. All authors have read and approved the final manuscript.
